# Self-compliance RRAM characteristics using a novel W/TaO_*x*_/TiN structure

**DOI:** 10.1186/1556-276X-9-292

**Published:** 2014-06-10

**Authors:** Siddheswar Maikap, Debanjan Jana, Mrinmoy Dutta, Amit Prakash

**Affiliations:** 1 Thin Film Nano Tech. Lab., Department of Electronic Engineering, Chang Gung University, 259 Wen-Hwa 1st Rd., Kwei-Shan, Tao-Yuan 333, Taiwan

**Keywords:** RRAM, Self-compliance, Resistive switching, TaO_*x*_, Non-linearity

## Abstract

Self-compliance resistive random access memory (RRAM) characteristics using a W/TaO_*x*_/TiN structure are reported for the first time. A high-resolution transmission electron microscope (HRTEM) image shows an amorphous TaO_*x*_ layer with a thickness of 7 nm. A thin layer of TiO_*x*_N_*y*_ with a thickness of 3 nm is formed at the TaO_*x*_/TiN interface, owing to the oxygen accumulation nature of Ti. This memory device shows 100 consecutive switching cycles with excellent uniformity, 100 randomly picked device-to-device good uniformity, and program/erase endurance of >10^3^ cycles. It is observed that the 0.6-μm devices show better switching uniformity as compared to the 4-μm devices, which is due to the thinner tungsten (W) electrode as well as higher series resistance. The oxygen-rich TaO_*x*_ layer at the W/TaO_*x*_ interface also plays an important role in getting self-compliance resistive switching phenomena and non-linear current-voltage (I-V) curve at low resistance state (LRS). Switching mechanism is attributed to the formation and rupture of oxygen vacancy conducting path in the TaO_*x*_ switching material. The memory device also exhibits long read endurance of >10^6^ cycles. It is found that after 400,000 cycles, the high resistance state (HRS) is decreased, which may be due to some defects creation (or oxygen moves away) by frequent stress on the switching material. Good data retention of >10^4^ s is also obtained.

## Background

Resistive random access memory (RRAM) is a potential candidate among all of the non-volatile memories because of its simple metal-insulator-metal (M-I-M) structure, fast switching speed, long endurance, stable data retention, low power operation, and high scalability potential [1-3]. Although some switching materials such as NiO [4,5], TiO_*x*_[6,7], HfO_*x*_[8-10], AlO_*x*_[11,12], and GdO_*x*_[13,14] have been reported, the TaO_*x*_ switching material is reported by few research groups [2,3,15-17]. Wei et al*.*[15] reported long endurance of >10^9^ cycles using Pt/Ta_2_O_5−*x*_/TaO_2−*x*_/Pt and Ir/Ta_2_O_5−*x*_/TaO_2−*x*_/Ir structures with an operation current of approximately 150 μA. Yang et al*.*[16] also reported long program/erase endurance of 10^10^ cycles using a Pt/TaO_*x*_/Ta structure with a high operation current. Lee et al. [2] reported the highest program/erase endurance of >10^10^ cycles using a Pt/Ta_2_O_5−*x*_/TaO_2−*x*_/Pt structure and that RRAM can be operated at a low current of <50 μA. Ninomiya et al*.*[18] reported that the operation current can be reduced to 80 μA by using a two-step formation in a Pt/Ta_2_O_5−*x*_/TaO_2−*x*_/Pt structure. In this case, the conducting filament can have a high oxygen vacancy density and thinner diameter, and data retention can also be improved. In our previous study, good resistive switching characteristics using a Ti interfacial layer in a W/TiO_*x*_/TaO_*x*_/W structure have been reported with an operation current of 80 μA. To get good resistive switching characteristics, almost all of the above structures need a higher formation voltage; most of them are not complementary metal-oxide-semiconductor (CMOS) compatible materials. To meet those requirements, a novel W/TaO_*x*_/TiN RRAM device has been investigated for the first time. All materials are CMOS compatible, and the self-compliance (SC) resistive switching phenomena with a low operation voltage of ±2.5 V are reported. This self-compliance property will have the capability of the memory device to control the current overshoot in a simple 1R configuration, which could be a good alternative for a one-transistor and one-resistor (1T1R) configuration.

In this study, self-compliance (<200 μA) bipolar resistive switching phenomena using a W/TaO_*x*_/TiN structure are reported under a low voltage of ±2.5 V. A high-resolution transmission electron microscope (HRTEM) image shows active RRAM size of 0.6 × 0.6 μm^2^. The thicknesses of TaO_*x*_ and TiO_*x*_N_*y*_ layers are approximately 7 and 3 nm, respectively. The memory device shows 100 consecutive bipolar resistive switching at low self-current compliance of <200 μA, good device-to-device uniformity, non-linear current-voltage (I-V) curve, and read endurance of >10^6^ cycles. It is found that the switching uniformity is better for the 0.6-μm devices as compared to the 4-μm devices, owing to the thinner tungsten (W) electrode as well as higher resistivity. Good data retention of >10^4^ s is also obtained.

## Methods

First, the SiO_2_ insulating layer with a thickness of 200 nm was grown on an 8-in. Si wafer. Then, the TiN as a bottom electrode (BE) was deposited by reactive sputtering. The thickness of TiN BE is approximately 250 nm. To isolate and fabricate the via-holes from 0.6 × 0.6 to 4 × 4 μm^2^, a low-temperature-deposited SiO_2_ layer with a thickness of approximately 150 nm was deposited on the TiN BEs. Different sizes of the via-holes and BE contacts were etched followed by lithography and etching processes. Photoresist (PR) was patterned, and the via-holes and top electrode (TE) regions were opened on the 8-in. wafers. Then, a wafer was broken into small pieces with each area of approximately 1 × 1.5 in. The TaO_*x*_ switching material with a thickness of approximately 7 nm was deposited by electron beam evaporation. Pure Ta_2_O_5_ shots were used for deposition. The deposition rate was 0.1 Å/s. The film became Ta:Ta_2_O_5_. Then, tungsten (W) TE with a thickness of approximately 400 nm was deposited by RF sputtering process. The deposition power and pressure were 100 W and 10 mTorr, respectively. Finally, lift-off was performed to get the final device. During measurement, the TiN BE was grounded and the voltage sweep was applied to the W TEs. Memory characteristics were measured by Agilent 4156C semiconductor parameter analyzer (Agilent Technologies, Santa Clara, CA, USA).

## Results and discussion

**Figure 1:**
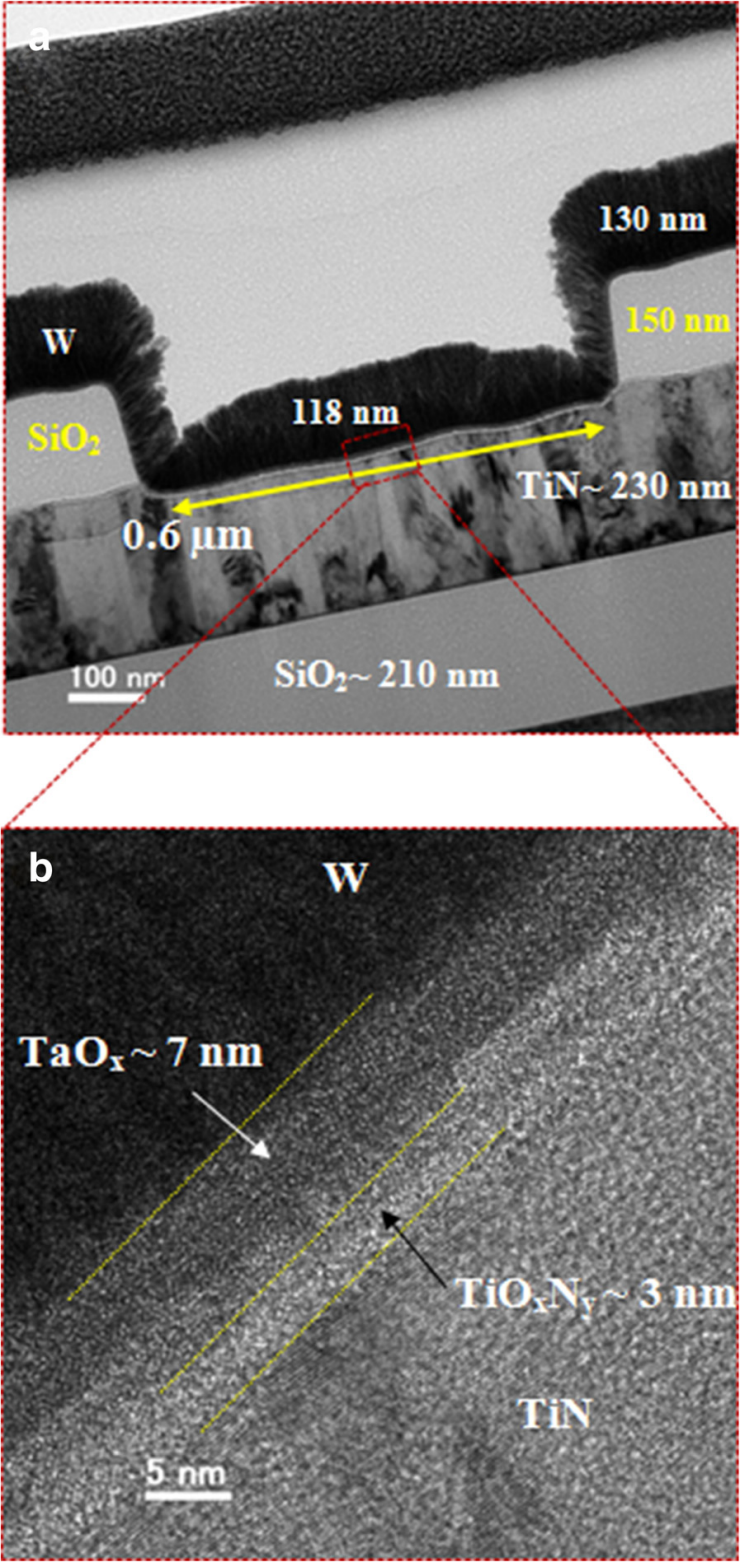
**TEM images of the RRAM device. (a)** A typical cross-sectional TEM image of a W/TaO_*x*_/TiN memory device. The device size is 0.6 × 0.6 μm^2^. **(b)** A HRTEM image showing the stacking layer of TaO_*x*_ and TiO_*x*_.

A typical cross-sectional transmission electron microscope (TEM) image of a RRAM device with a size of approximately 0.6 × 0.6 μm^2^ is shown in Figure 1a. The deposition recipe of W TE was approximately 150 nm. However, the thicknesses of W TE are 118 and 130 nm inside and outside of the via-hole regions, respectively, although it is smaller on the sidewall of approximately 50 nm. However, this issue is not present for larger size (4 × 4 μm^2^) devices. This suggests that via-hole filling of W TE is easier for the larger size than for the smaller size devices. Thus, because of thickness-dependent W TE resistivity as well as device size, the self-compliance resistive switching characteristics differ. The electrical resistivity of W TE is higher for the smaller size devices than for the larger size devices. In this case, all electrical measurements were done with a W TE deposition recipe of approximately 400 nm. This thickness will be maintained for the larger size devices, and it will be smaller for the smaller size devices and electrical resistivity will be increased as well. Figure 1b shows a HRTEM image of the W/TaO_*x*_/TiN structures. The TaO_*x*_ film is amorphous. The thicknesses of TaO_*x*_ and TiO_*x*_N_*y*_ layers are approximately 7 and 3 nm, respectively. This is due to the fact that Ti is more reactive with O_2_ (Gibb's free energy −883.32 kJ/mol at 300 K [19,20]) resulting in the formation of a TiO_2_ layer, i.e., TiO_*x*_N_*y*_. It might be possible that during Ta_2_O_5_ deposition, Ti takes oxygen from Ta_2_O_5_, forms a TiO_*x*_N_*y*_ layer, and makes a defective TaO_*x*_ switching material. However, the TiO_*x*_N_*y*_ layer will be more electrically conducting than the TaO_*x*_ layer, and the conducting filament formation/rupture can happen inside the TaO_*x*_ switching layer. Due to a series of TiO_*x*_N_*y*_ layers with TaO_*x*_, enhanced resistive switching memory characteristics could be observed as discussed later.

**Figure 2:**
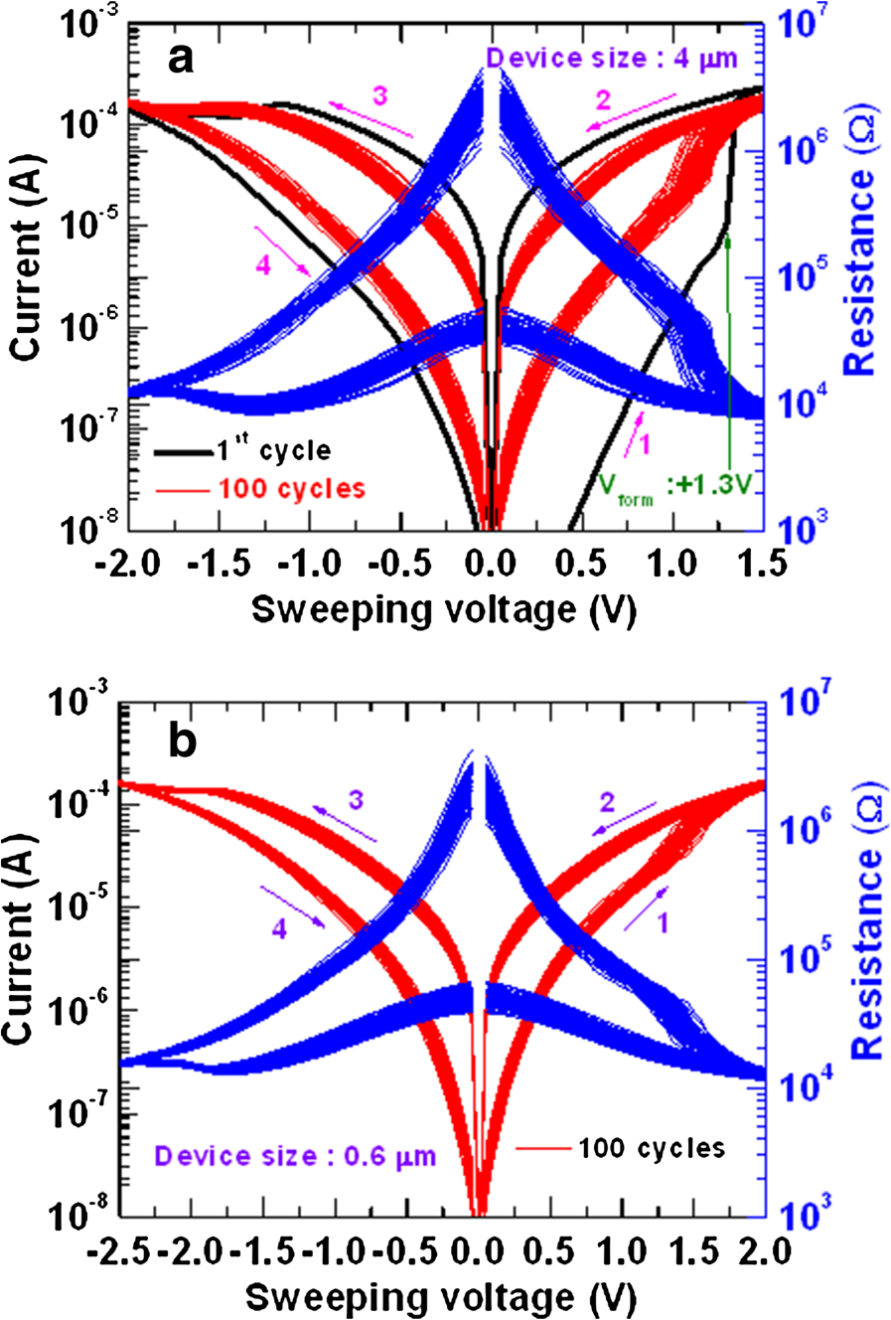
**Current**-**voltage and resistance**-**voltage switching characteristics with different device sizes.** Current-voltage and corresponding resistance-voltage characteristics of the W/TaO_*x*_/TiN memory devices with different device sizes of **(a)** 4 × 4 and **(b)** 0.6 × 0.6 μm^2^. The memory device performs 100 consecutive cycles of self-compliance bipolar resistive switching under a small operating voltage of ±2.5 V. Repeatable switching cycles are observed. The voltage-sweeping directions are shown by arrows 1 to 4.

**Figure 3:**
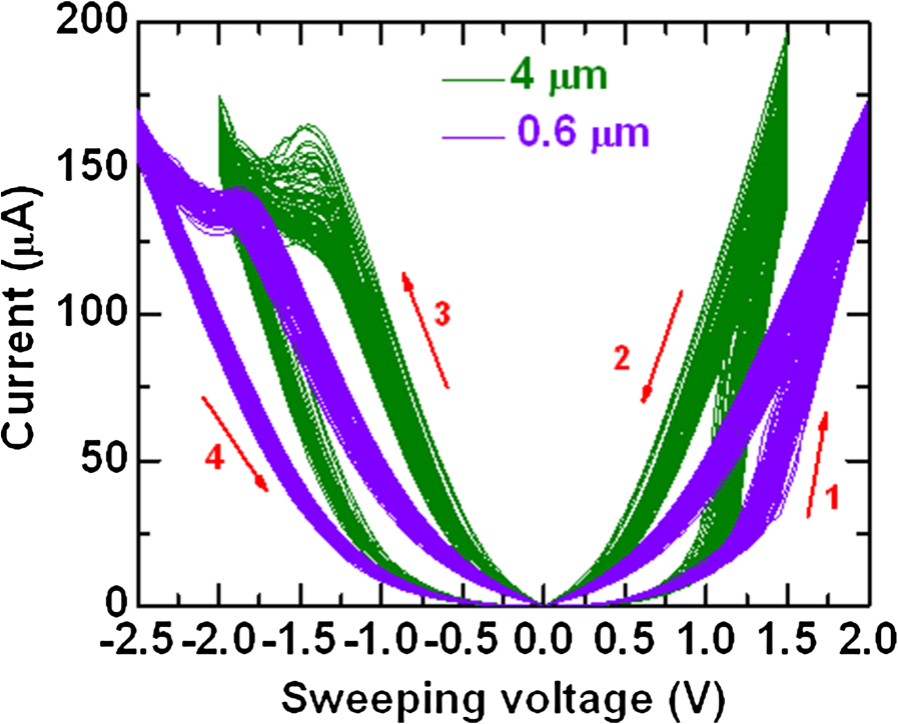
**One hundred consecutive switching cycles with linear scale**. Non-linear I-V curves are observed. The voltage-sweeping directions are shown by arrows 1 to 4.

Figure 2 exhibits self-compliance bipolar current-voltage (I-V) and corresponding resistance-voltage (R-V) characteristics of the W/TaO_*x*_/TiN RRAM devices. The voltage-sweeping directions are shown by arrows 1 to 4. The device sizes were 4 × 4 μm^2^ (Figure 2a) and 0.6 × 0.6 μm^2^ (Figure 2b). A small formation voltage (*V*_form_) of 1.3 V is needed to form the conducting filament, as shown in Figure 2a. After the first RESET operation, the memory devices show 100 consecutive switching cycles at a low self-compliance (SC) current of 139 to 196 μA with a small operation voltage of +1.5/−2 V for the 4-μm devices and 136 to 176 μA with an operation voltage of +2/−2.5 V for the 0.6-μm devices. The SET voltages are slightly varied from 1.0 to 1.2 V and 1.2 to 1.5 V for the 4- and 0.6-μm devices, respectively. Both high resistance state (HRS) and low resistance state (LRS) are varied with 100 cycles from 0.83 to 3.47 M and 28 to 55 kΩ, and 0.97 to 3.12 M and 37.4 to 64.7 kΩ at a read voltage (*V*_read_) of 0.1 V for the 4- and 0.6-μm devices, respectively. The RESET voltages and currents are found to be −1.45 V and approximately 165 μA, and −1.85 V and approximately 144 μA for the 4- and 0.6-μm devices, respectively. In addition, non-linearity of the I-V curves at LRS for the 0.6-μm devices is better than that for the 4-μm devices (Figure 3). The 0.6-μm devices show higher values of SET/RESET voltages, better switching uniformity in cycles-to-cycles, better non-linearity, and lower SC operation, owing to the higher series resistivity to W TE than that of the 4-μm devices. However, all sizes of RRAM devices are operated with a small voltage of ±2.5 V.

**Figure 4:**
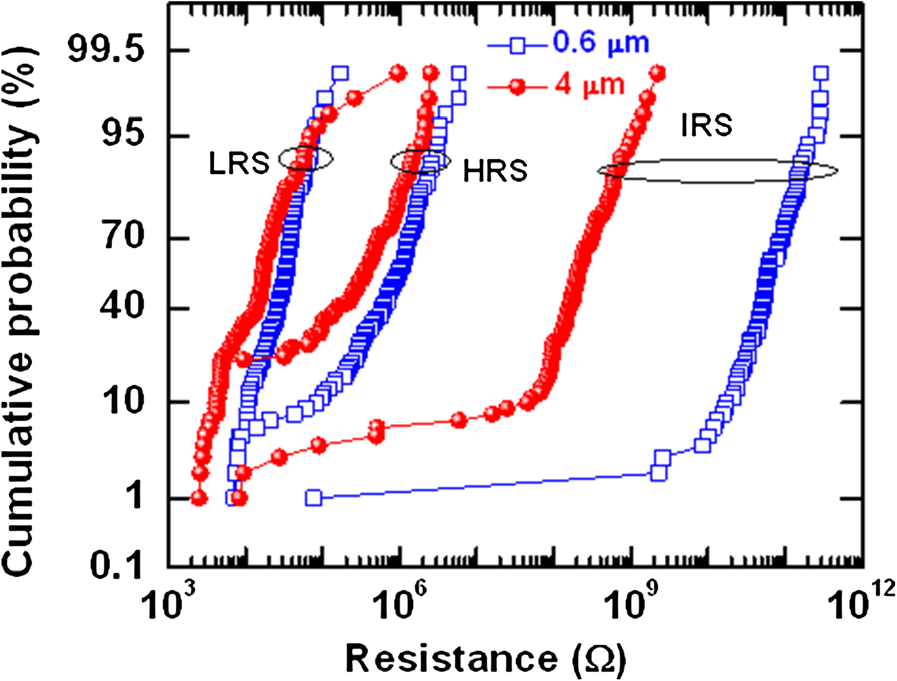
**Cumulative probability.** IRS, HRS, and LRS of 100 devices are plotted. The 0.6-μm device shows slightly better uniformity.

**Figure 5:**
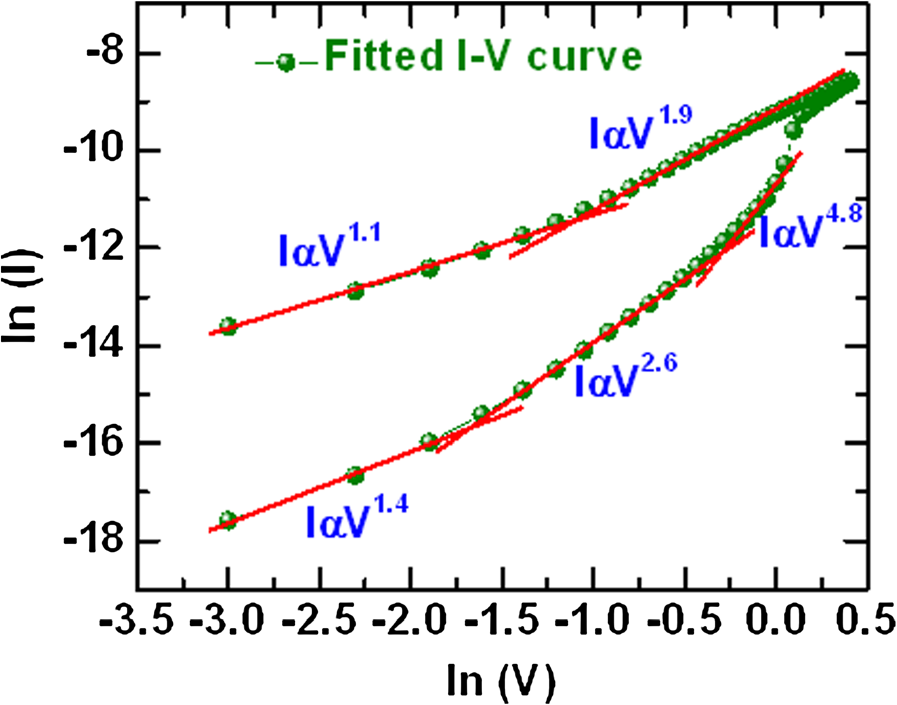
**I**-**V curve fitted in log**-**log scale.** Both HRS and LRS show a trap-controlled space-charge-limited current conduction (TC-SCLC) mechanism. The device size is 4 × 4 μm^2^.

To investigate the switching uniformity for high-density memory application, more than 100 devices were randomly measured for both the 4- and 0.6-μm devices, as shown in Figure 4. The cumulative probability of initial resistance state (IRS) for the 0.6-μm devices is higher than that for the 4-μm devices (56.6 G vs. 189.5 MΩ at 50% probability). This suggests that a larger size device has more defects than a smaller size device, which may cause lower IRS. However, some devices have shown failure and could be improved in the future. Except for a few, memory devices show excellent device-to-device uniformity with a yield of approximately 90%. The average values (standard deviation) of HRS and LRS for the 0.6-μm devices are found to be 1.1 (111.39) M and 33.6 (23.49) kΩ, while those for the 4-μm devices are found to be 486.6 (59.25) M and 24.83 (97.6) kΩ, respectively. This suggests that the RRAM devices show acceptable uniformity. Especially, improved uniformity with higher LRS is observed for the 0.6-μm devices, owing to the thinner W TE as well as higher series resistivity. To realize the current conduction mechanism, the I-V curve was fitted in a log-log scale as shown in Figure 5. Slope values of LRS are 1.1 (IαV^1.1^) and 1.9 (IαV^1.9^) whereas slope values of HRS are 1.4 (IαV^1.4^), 2.6 (IαV^2.6^), and 4.8 (IαV^4.8^). This suggests that the current conduction mechanism of our memory device is dominated by a trap-controlled space-charge-limited current conduction mechanism. Oxygen vacancies might be serving as the trap sites. The switching mechanism is ascribed to the formation and rupture of oxygen vacancy conducting path in the TaO_*x*_ switching material under external bias. When a positive bias is applied to the TE, Ta-O bonds break and O^2−^ ions migrate towards the TE/TaO_*x*_ interface and generate an oxygen-rich TaO_*x*_ layer at the interface, leaving behind oxygen vacancies to form the conducting path, and the RRAM devices switch from HRS to LRS. This electrically formed interfacial oxygen-rich layer behaves like series resistance at the interface [21] which opposes to form the continuous filament. The discontinuous filament formation due to the oxygen-rich layer at the TE interface might cause the non-linear behavior of the I-V curve at LRS and self-compliance phenomena of our memory device as well.

**Figure 6:**
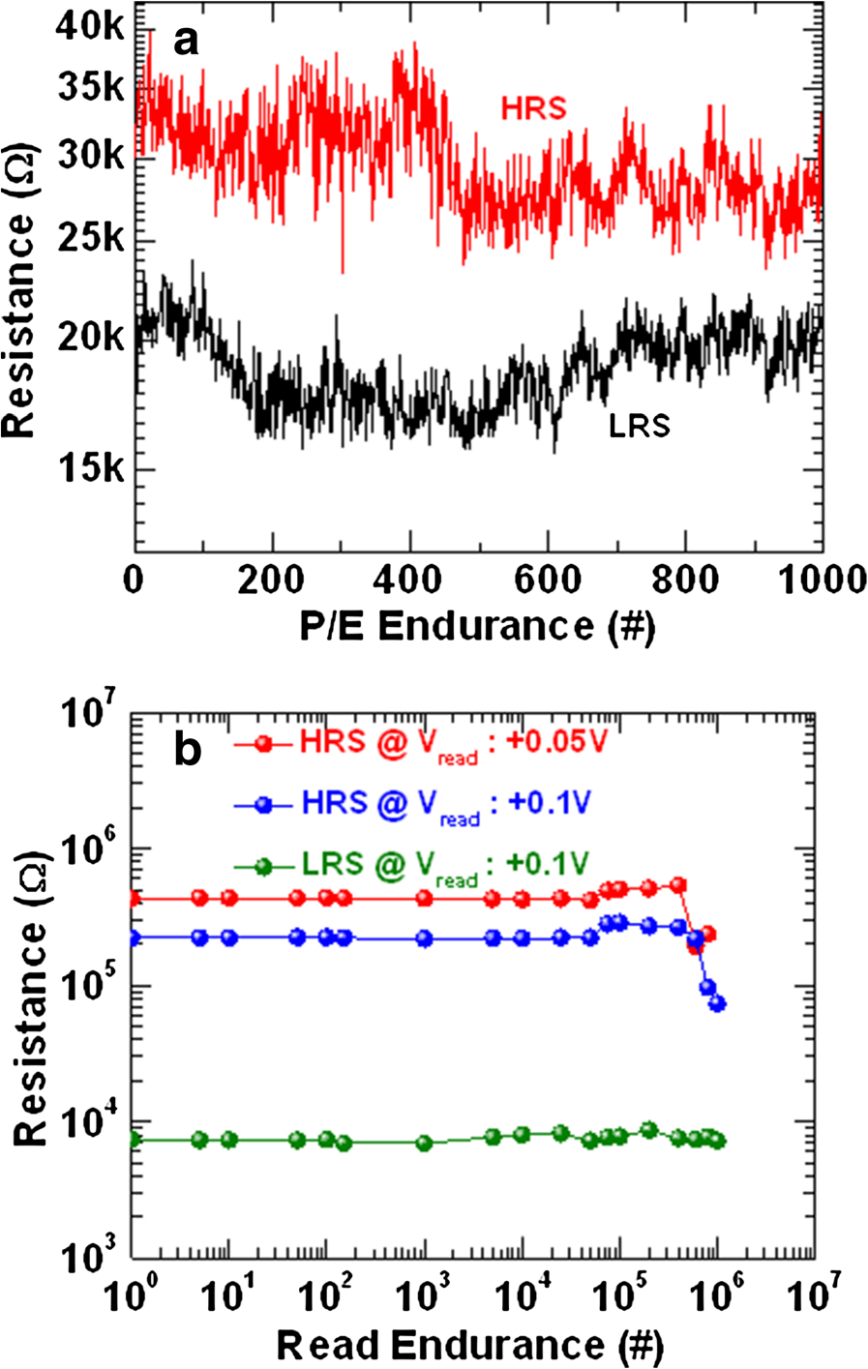
**Endurance characteristics. (a)** P/E endurance of >10^3^ cycles and **(b)** long read pulse endurance of >10^6^ cycles of our novel W/TaO_*x*_/TiN memory device. The device size is 4 × 4 μm^2^.

**Figure 7:**
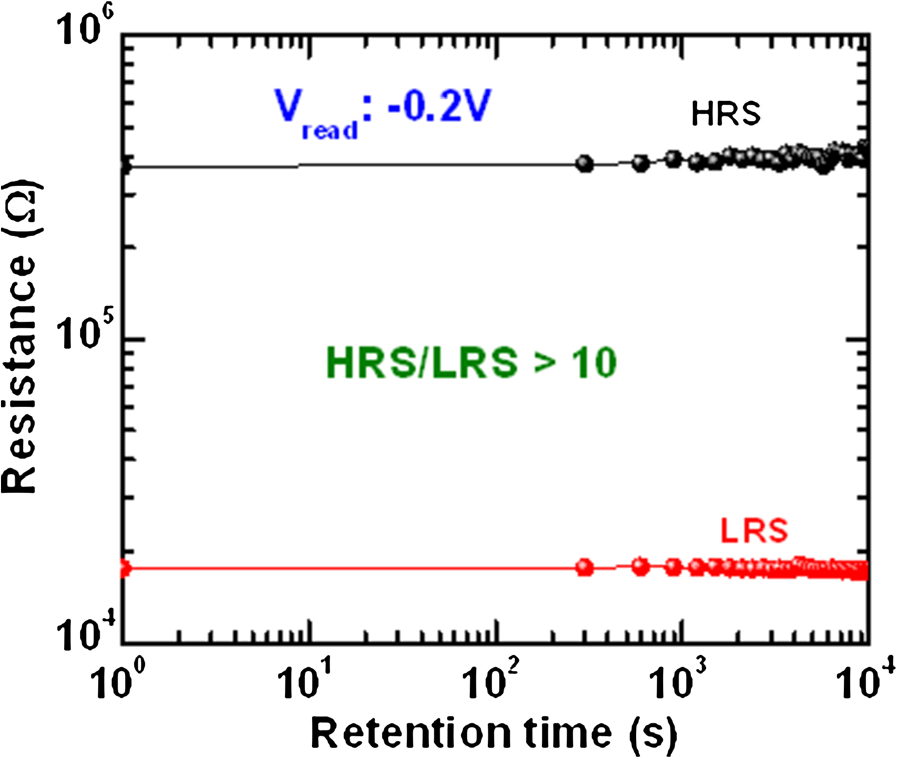
**Data retention characteristics.** Good data retention of >10^4^ s of our W/TaO_*x*_/TiN memory device. An acceptable resistance ratio of >10 is obtained after 10^4^ s.

Figure 6a exhibits the program/erase (P/E) endurance of >1,000 cycles of the W/TaO_*x*_/TiN RRAM device. The device size is 4 × 4 μm^2^. Every cycle data was captured during measurement. The P/E voltages were +2/−2.2 V. Both HRS and LRS were read out at +0.1 V, and pulse width was 500 μs. The P/E cycles are not stable as we expected. Further study is needed to obtain stable P/E cycles. Long read pulse endurance of >10^6^ cycles is shown in Figure 6b. In this case, stress pulse width was 500 μs and read pulse width was 10 μs. Stable LRS is obtained at a *V*_read_ of 0.1 V. Due to the strong conducting filament formation, stable LRS is observed under random read pulse. For LRS only, it took a long measurement time of approximately 3 days. On the other hand, the data retention is quite good after programming the device. The HRS was read out at two different *V*_read_'s of +0.1 and +0.05 V. Stable HRS is observed up to 400,000 cycles, and the HRS is decreased with pulse numbers. This may be due to defects creation during continuous stress on the TaO_*x*_ switching layer or the migration of oxygen ions due to heating effects. Further study is needed to improve P/E endurance and instability of read pulse endurance of HRS after long cycles. However, a resistance ratio of >10 is obtained after 10^6^ cycles. Our memory device also performs good data retention of >10^4^ s as shown in Figure 7. The read voltage for both HRS and LRS was −0.2 V. An acceptable resistance ratio of >10 is observed after a retention time of 10^4^ s. This RRAM device is very useful for nanoscale non-volatile memory application.

## Conclusions

One hundred consecutive switching cycles in the W/TaO_*x*_/TiN structures under self-compliance (<200 μA) and low-voltage operation of ±2.5 V are obtained. The thicknesses of TaO_*x*_ and TiO_*x*_N_*y*_ layers are 7 and 3 nm, respectively, which are observed by HRTEM. The RRAM device sizes are also confirmed by TEM. Our memory device shows good switching characteristics at low self-current compliance with tight distribution of HRS/LRS, excellent device-to-device uniformity, and program/erase endurance of >1,000 cycles. The smaller size devices show better switching characteristics and uniformity as compared to the larger size devices, owing to the thinner W electrode as well as higher series resistance. Interfacial oxygen-rich TaO_*x*_ layer acts as a series resistance to control the resistive switching characteristics which may also cause the self-compliance resistive switching behavior and non-linear I-V curve at LRS. Switching mechanism is based on the formation and rupture of oxygen vacancy conducting path in the TaO_*x*_ switching material. The memory device also exhibits a long read endurance of >10^6^ cycles and good data retention of >10^4^ s with a resistance ratio >10. Therefore, this self-compliant W/TaO_*x*_/TiN device will have great potential for future non-volatile memory application.

## Competing interests

The authors declare that they have no competing interests.

## Authors' contributions

DJ and AP fabricated the RRAM devices under the instruction of SM. MD measured the devices under the instruction of SM. SM also measured the devices. AP helped in understanding the switching characteristics. All the authors contributed to the revision of the manuscript, and they approved it for publication.
